# Repeated mass strandings of Miocene marine mammals from Atacama Region of Chile point to sudden death at sea

**DOI:** 10.1098/rspb.2013.3316

**Published:** 2014-04-22

**Authors:** Nicholas D. Pyenson, Carolina S. Gutstein, James F. Parham, Jacobus P. Le Roux, Catalina Carreño Chavarría, Holly Little, Adam Metallo, Vincent Rossi, Ana M. Valenzuela-Toro, Jorge Velez-Juarbe, Cara M. Santelli, David Rubilar Rogers, Mario A. Cozzuol, Mario E. Suárez

**Affiliations:** 1 Department of Paleobiology, National Museum of Natural History, Smithsonian Institution, PO Box 37012, Washington, DC 20013, USA; 2 Department of Mineral Sciences, National Museum of Natural History, Smithsonian Institution, PO Box 37012, Washington, DC 20013, USA; 3 Department of Mammalogy, Burke Museum of Natural History and Culture, Seattle, WA 98195, USA; 4 Department of Paleontology, Burke Museum of Natural History and Culture, Seattle, WA 98195, USA; 5 Red Paleontológica, Laboratorio de Ontogenia y Filogenia, Departamento de Biología, Facultad de Ciencias, Universidad de Chile, Las Palmeras, Santiago 3425, Chile; 6 John D. Cooper Archaeological and Paleontological Center, Department of Geological Sciences, California State University, Fullerton, CA 92834, USA; 7 Departamento de Geología, Facultad de Ciencias Físicas y Matemáticas and Andean Geothermal Center of Excellence, Universidad de Chile, Plaza Ercilla 803, Santiago, Chile; 8 Digitization Program Office 3D Lab, Office of the Chief Information Officer, Smithsonian Institution, Landover, MD 20785, USA; 9 Laboratorio de Ecofisiología, Departamento de Ecología, Facultad de Ciencias, Universidad de Chile, Las Palmeras, Santiago 3425, Chile; 10 Florida Museum of Natural History, University of Florida, Gainesville, FL 32611, USA; 11 Área Paleontología, Museo Nacional de Historia Natural, Casilla 787, Santiago, Chile; 12 Departamento de Zoologia, Instituto de Ciências Biológicas, Universidade Federal de Minas Gerais, Belo Horizonte, Minas Gerais, Brazil

**Keywords:** taphonomy, strandings, fossil record, harmful algal blooms

## Abstract

Marine mammal mass strandings have occurred for millions of years, but their origins defy singular explanations. Beyond human causes, mass strandings have been attributed to herding behaviour, large-scale oceanographic fronts and harmful algal blooms (HABs). Because algal toxins cause organ failure in marine mammals, HABs are the most common mass stranding agent with broad geographical and widespread taxonomic impact. Toxin-mediated mortalities in marine food webs have the potential to occur over geological timescales, but direct evidence for their antiquity has been lacking. Here, we describe an unusually dense accumulation of fossil marine vertebrates from Cerro Ballena, a Late Miocene locality in Atacama Region of Chile, preserving over 40 skeletons of rorqual whales, sperm whales, seals, aquatic sloths, walrus-whales and predatory bony fish. Marine mammal skeletons are distributed in four discrete horizons at the site, representing a recurring accumulation mechanism. Taphonomic analysis points to strong spatial focusing with a rapid death mechanism at sea, before being buried on a barrier-protected supratidal flat. In modern settings, HABs are the only known natural cause for such repeated, multispecies accumulations. This proposed agent suggests that upwelling zones elsewhere in the world should preserve fossil marine vertebrate accumulations in similar modes and densities.

## 1. Introduction

During the past approximately 50 Myr, marine mammals evolved in ocean ecosystems that have undergone global changes in sea level, temperature, productivity and ocean circulation [1–5]. Within this time frame, multiple marine mammal lineages evolved from trophic obscurity (i.e. terrestrial ancestry, with little influence on ocean ecosystems) to ecological dominance in marine food webs [6–10]. Understanding how marine mammals, such as cetaceans, pinnipeds and sirenians, ascended to become apex consumers in marine food webs [11] requires data from the fossil record. Palaeobiologists have used counts of fossil species [2,4,5,12] to outline evolutionary changes in richness at the scale of geologic time [13], especially during episodes of major climatic changes [3,4]. These latter studies investigated evolutionary causes and responses over protracted, diachronic time frames. However, testing ecological interactions requires diversity datasets from synchronic snapshots at specific scales that account for time-averaging, sampling density and other metrics of diversity, such as abundance [14]. This latter goal is a challenge because the marine mammal fossil record consists mostly of singleton occurrences [13] and not dense accumulations.

Obtaining ecological snapshots of large mobile predators such as marine mammals is logistically difficult because their life-history traits (e.g. long life, low fecundity, large range) have broad temporal and geographical parameters [6–9]. Palaeoecologists working with terrestrial mammal and marine invertebrate communities have discovered that sampling diversity with increased temporal- and spatial-averaging generates death assemblage datasets that compare well with living communities [15–18]. Recently, Pyenson [19,20] demonstrated that death assemblages of modern cetaceans (e.g. strandings) faithfully record ecological snapshots of living communities at temporal and spatial scales commensurate with their macroecology, which suggests that certain fossil assemblages might retain similarly faithful ecological data. Marine mammal mass strandings are well recorded in historical times [19,20], but the putative cases from the fossil record cannot be linked to a particular causal mechanism [21,22], especially without a better understanding of the taphonomic mechanisms that both preserve and prevent fossil marine mammal material from entering the sedimentary record [23]. Here, we describe an unusual accumulation of fossil marine vertebrates from the Late Miocene of Chile that provides unique insights into the mechanisms that preserve dense deposits of marine mammal material, and the oceanographic processes responsible for their origin.

## 2. Material and methods

### (a) Locality

**Figure 1.**
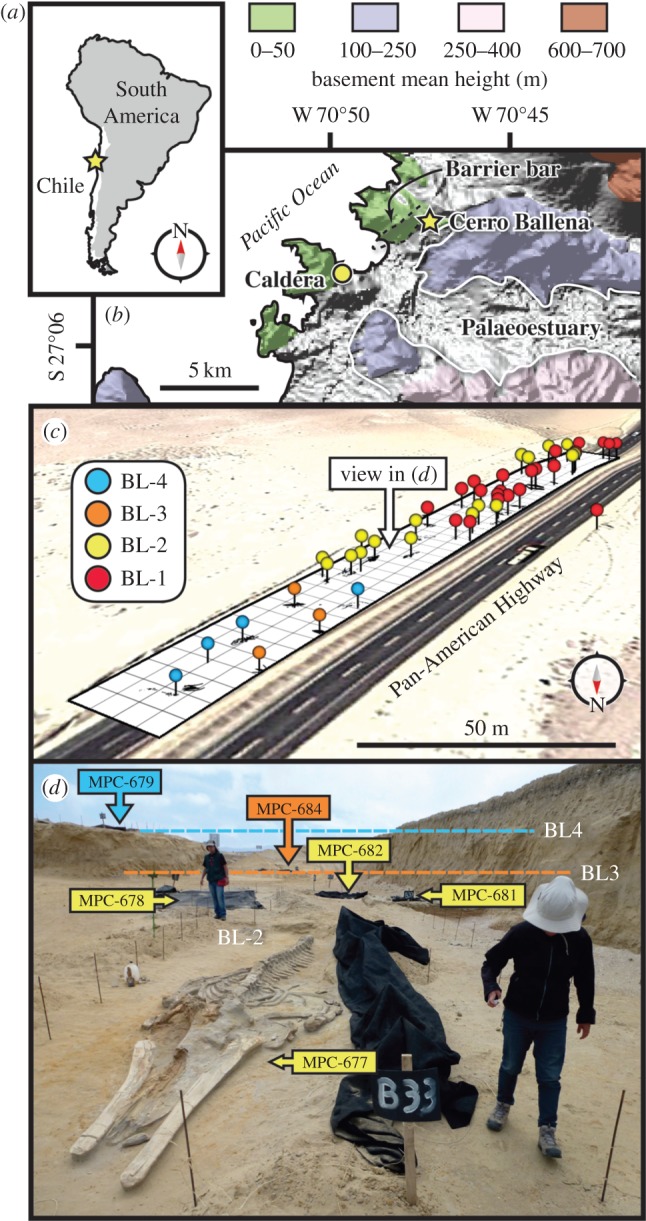
Locality and geographical information for Cerro Ballena showing (*a*) South America with (*b*) the palaeocoastline of the Caldera Basin outlined over NASA Shuttle Radar Topography Mission data; (*c*) quarry map showing specimen positions and colour-coded stratigraphic layer, created in Google Earth; (*d*) view of the quarry and the top three bone-bearing layers (BL2–4), facing northeast, from MPC 677 *in situ*. See http://cerroballena.si.edu and the electronic supplementary material for more details.

**Figure 2.**
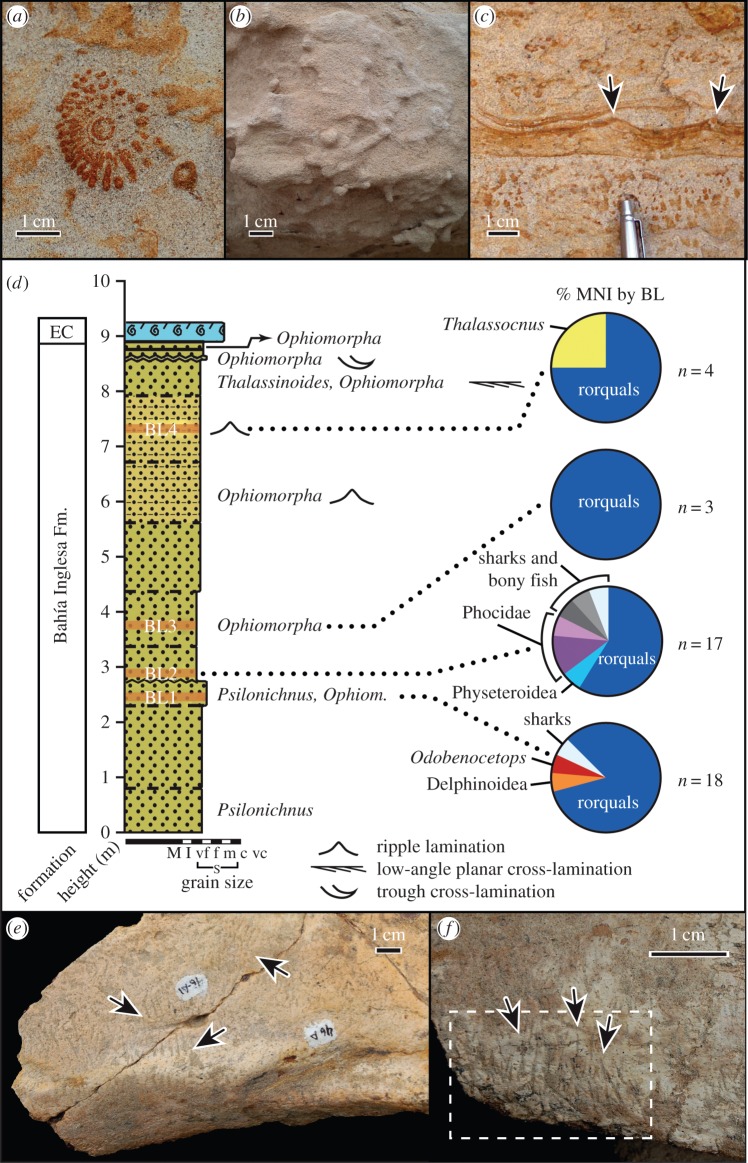
Stratigraphic and sedimentological data from Cerro Ballena. (*a*) Unidentified, iron-stained traces resembling algal growth structures; (*b*) *Psilonichnus*, a supratidal trace fossil; (*c*) iron-stained tuft-like forms resembling algae, and possible algal mats covering wave ripples (indicated with arrows), with pen for scale and (*d*) stratigraphic column, with vertebrate diversity data expressed as percentage of the MNI from each bone-bearing layer (BL1–4). (*e,f*) Crab feeding traces on the skull bones of MPC 662, from BL-1. c, coarse grained; EC, Estratos de Caldera; f, fine grained; I, siltstone; M, mudstone; m, medium grained; S, sandstone; vc, very coarse grained; vf, very fine grained.

From 2010 to 2012, road expansion along the Pan-American Highway in Atacama Region of Chile (figure 1) opened a 20 × 250 m quarry at a site, called Cerro Ballena (27°02′31.51″ S, 70°47′42.18″ W), which revealed over 40 complete and partial marine mammal skeletons, along with isolated remains of other marine vertebrates. A road-cut stratigraphic profile of the site exposes approximately 9 m of fine to very fine-grained sandstones belonging to the Bahía Inglesa Formation [24–27], unconformably overlain by a Pleistocene transgressive–regressive marine terrace sequence [28]. Cerro Ballena is located too far north for stratigraphic correlation with other reported localities of the Bahía Inglesa Formation [28], or any recognized members that have been proposed previously ([26] and see the electronic supplementary material). Within approximately 8 m of the formation at Cerro Ballena, four different bone-bearing levels (Bone Levels 1–4; BL1–BL4) produced articulated and associated marine mammal fossils (figures 1 and 2). The quarry, which is now paved over, represents only a small portion of the fossiliferous levels, with geologic maps of the unit indicating a local aerial extent of approximately 2 km^2^ (see the electronic supplementary material).

### (b) Geologic age

The general region surrounding Caldera contains fossiliferous marine sediments belonging to the Mio-Pliocene age Bahía Inglesa Formation [25], which has produced an extensive list of fossil marine vertebrates [26–28]. We did not find any biostratigraphically relevant invertebrate and microfossil indicators in strata at Cerro Ballena, although there were two biostratigraphically useful vertebrate fossils from Cerro Ballena that also occur in the Mio-Pliocene Pisco Formation of Peru: aquatic sloths (*Thalassocnus*) and sharks (*Carcharodon*). Isolated elements of aquatic sloths from Cerro Ballena are referred to the species *Thalassocnus natans* [29]. Both *T. natans* and *Carcharodon hastalis* from Cerro Ballena are correlated with the El Jahuay (ELJ) and Montemar Horizon (MTM) horizons in the Sacaco Basin of Peru [30]. In turn, this yields upper and lower bounds on the age of this unit of the Bahía Inglesa Formation at Cerro Ballena as 9.03–6.45 Ma, following [31] (see the electronic supplementary material). The overlap in stratigraphic range between these two taxa implies that the strata in Cerro Ballena were deposited at a time period in between the deposition of the ELJ and MTM horizons of the Pisco Formation. Thus, we infer a Late Miocene age (or Late Tortonian to Early Messinian stage) for the Bahía Inglesa Formation part of the section at Cerro Ballena, which coincided with a rise in sea level caused by transgressive–regressive cycling and tectonic subsidence along this part of the coastline [32].

### (c) Depositional environment and sedimentology

In the road-cut section of the Bahía Inglesa Formation at Cerro Ballena, we measured a stratigraphic section, noted sedimentary structures and observed invertebrate trace fossils (figure 2*a–c*).

Sediment samples collected from BL1–4 were freshly excavated and covered in optically clear resin (Epo-Tek 301, Epoxy Technology, Billerica, MA, USA). Embedded samples were then cut and thin-sectioned for scanning electron microscopy and electron spectroscopy using an FEI Nova NanoSEM 600 under low vacuum with the gaseous analytical detector for imaging and an energy dispersive X-ray spectroscopy detector (ThermoFisher) for geochemical analysis. Samples were either placed directly on carbon-tape or embedded in epoxy and thin-sectioned, and left uncoated for SEM and EDS characterization (see the electronic supplementary material, figure S1). Light microscopy was performed using an Olympus BX51 microscope with a Chameleon digital video camera.

### (d) Capturing, processing and rendering three-dimensional digital datasets

**Figure 3.**
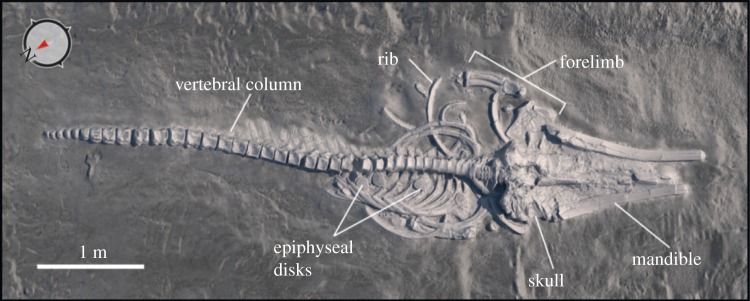
Orthogonal digital three-dimensional polygon model of the most complete fossil rorqual specimen at Cerro Ballena, MPC 677. True north indicated by arrow. See http://cerroballena.si.edu and the electronic supplementary material for more details.

**Figure 4.**
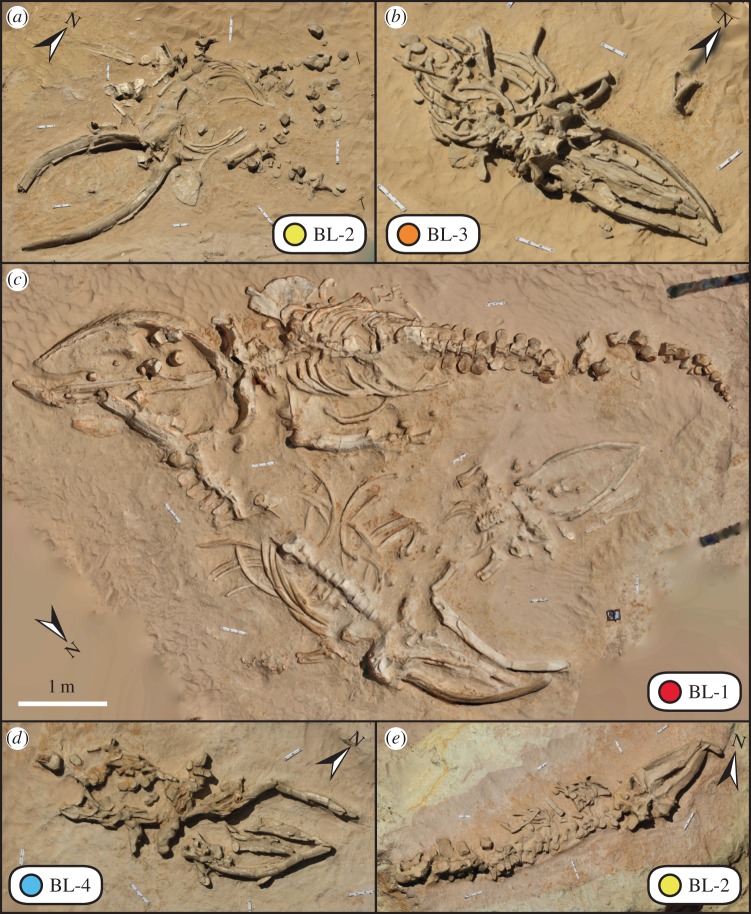
High dynamic range images of orthogonal three-dimensional point clouds capturing adult and juvenile fossil rorqual skeletons from Cerro Ballena. (*a*) MPC 678; (*b*) MPC 684; (*c*) over-lapping adult and juvenile specimens, clockwise MPC 666, 665 and 667; (*d*) MPC 685 and (*e*) MPC 675. Small-scale bars 20 cm, large-scale bars 30 cm. True north indicated by arrow, and stratigraphic layer noted by bone-bearing level number. See http://cerroballena.si.edu and the electronic supplementary information for more details and source data.

Under time-sensitive and salvage circumstances, we documented *in situ* skeletal remains using three-dimensional digital tools, before they were collected for study and care at their repositories (see the electronic supplementary material). Photogrammetry and computer vision datasets for fossil rorquals were captured with 20 and 30 cm aluminium scale bars and metal markers (to assess line of sight and control for coverage quality) on a Canon 5D with multiple lenses, and geotagged using a Garmin Etrex GPS. We also used Munsell colour charts for colour calibration, accuracy and downstream correction in photography editing software packages. Raw digital datasets were processed into coherent models by aligning datasets, cleaning up noise and removing redundant data using Geomagic v. 2012, Polyworks v. 12.0 and Zbrush v. 4R3 for the very large dataset of MPC (Museo Paleontologico de Caldera) 677. Direct Dimensions, Inc. (Owings Mills, MD, USA) aligned the laser arm dataset using Geomagic, and the model was then retopologized using Zbrush for MPC 677, creating an orthogonal digital rendering from three-dimensional polygon data (figure 3). URC Ventures (Redmond, WA, USA) created orthogonal renderings from three-dimensional point cloud datasets (figure 4) by aligning and retopologizing point cloud data (see the electronic supplementary material, figure S2). The resultant three-dimensional datasets provided sub-centimetre accuracy, and full resolution texture-mapped imagery is available at http://cerroballena.si.edu.

## 3. Results and discussion

Taphonomic analysis of the site reveals several features that are directly comparable to modern marine mammal mass strandings. First, the site preserves multiple species of marine mammals, dominated by abundant skeletons (MNI = 31; table 1) of large baleen whales (clade Balaenopteridae or rorquals) that are likely all from the same species (see the electronic supplementary materials), and encompass a range of ontogenetic stages, from calves to mature individuals (figures 3 and 4). Other marine mammal species include: (i) at least two different phocid seals (*Acrophoca*, and a new morphotype); (ii) an extinct species of sperm whale (*Scaldicetus* morphotype); (iii) a walrus-like toothed whale (*Odobenocetops*) and (iv) an aquatic sloth (*T. natans*) (tables 1 and 2; see the electronic supplementary material, figures S3–S7). Second, we devised a simple, three-stage categorization to capture the range of marine vertebrate taphonomy at Cerro Ballena: (Stage 1) articulated, either completely or mostly; (Stage 2) disarticulated, but associated elements and (Stage 3) isolated, separated elements. Non-cetacean vertebrates consist of associated, semi-articulated and/or disarticulated skeletal material (Stages 2 and 3). By contrast, rorqual skeletons included many fully articulated, intact and nearly complete skeletons (Stage 1), along with disarticulated skeletons with low skeletal scatter (i.e. less than the distance of their body length; figure 3; table 2; and see the electronic supplementary material, tables S1–S11). Third, rose diagrams of the rorquals’ skeletons long axis orientation (i.e. vertebral column) reveal that they are orthogonal to current flow in each level, analogous to body orientation patterns observed for some modern mass strandings ([33]; see the electronic supplementary material, figure S8 and table S4). Lastly, rorquals occur mostly ventral up, across all BLs (table 2). The dominance of ventral up carcasses, combined with their high articulation and long axis orientation, is a strong sign that they washed in dying or dead and were then buried [34,35].

**Table 1.** Diversity of fossil marine vertebrates at Cerro Ballena, with minimum number of individuals (MNI) by bone-bearing level (BL) and with range of skeletal articulation stages. This tabulation does not include 11 additional, unidentified large cetacean skeletons (see the electronic supplementary material, figures S3–S7 and S9).

clade	taxon	BL occurrence	total MNI	articulation
Mysticeti	Balaenopteridae	BL 1–4	31	Stages 1–3
Phocidae	*Acrophoca* sp.	BL 2	2	Stages 2 and 3
Elasmobranchii	*Carcharodon hastalis*	BL 1, 2	2	Stage 3
Odontoceti	Delphinoidea	BL 1	1	Stage 3
Odontoceti	Physeteroidea	BL 2	1	Stages 2 and 3
Odontoceti	*Odobenocetops* sp.	BL 1	1	Stage 2
Phocidae	Phocidae n. gen.	BL 2	1	Stage 3
Nothrotheriidae	*Thalassocnus natans*	BL 4	1	Stage 3
Osteichythes	Istiophoridae	BL 2	1	Stage 3
Osteichythes	Xiphiidae	BL 2	1	Stage 3

**Table 2.** Taphonomic attributes of fossil rorqual skeletons at Cerro Ballena, ranked stratigraphically by BL. Number of individual specimens (NISP) is scored for percentage oriented ventral up, skeletal articulation and scatter and total length (TL). See the electronic supplementary material, tables S1–11.

BL level	% ventral up	NISP	dominant mode(s) of articulation	average scatter (m)	NISP for scatter	average TL (m)	NISP for TL
BL-4	33	3	Stage 2	2.77	3	8.62	3
BL-3	67	3	Stage 1	2.21	3	7.63	2
BL-2	67	6	Stages 1 and 3	2.80	7	7.43	7
BL-1	92	12	Stage 1	3.45	13	7.97	9
average	75			2.83		7.91	

The dominance of fossil rorqual skeletons at Cerro Ballena, across all bone-bearing levels, evokes a modern stranding event, whereby many individual cetaceans are beachcast, either dead or alive. Mass stranding events for socially gregarious species of toothed whales are well documented [36], but mass strandings for rorquals are rare. The most compelling analogue is a rorqual mass stranding of 14 humpback whales (*Megaptera novaeangliae*) over the course of five weeks along approximately 50 km of coastline around Cape Cod, MA, USA in 1987–1988 [35]. This assemblage included males, females and one calf, whose necropsies showed no signs of trauma or predation. Tissue assays of Atlantic mackerel (*Scomber scombrus*), from stomach contents, revealed high concentrations of saxitoxins, which are dinoflagellate neurotoxins. This evidence, along with the documented aberrant behaviour of one of the dying whales, and the geographical and temporal spans of the event, pointed to the previously unrecognized trophic transfer of major algal toxins. Since then, other cases of harmful algal blooms (HABs) involving marine mammals have been reported at similar geographical and temporal scales [37–41].

The assemblage at Cerro Ballena shares specific features with HAB-mediated mass strandings. These similarities help delimit the cause of death and the factors that have driven their concentration and preservation at this site. The presence of repeated, multispecies assemblages argues for a taxonomically broad death mechanism, such as HABs. The proximity of many specimens, including juvenile and adult rorqual skeletons in direct contact or few metres apart (figure 4*c*), along with different marine vertebrate taxa approximately 10 m apart, suggests strong post-mortem spatial focusing, prior to burial at each level (figure 1*c*). Intraspecific and interspecific taphonomic variation does not eliminate this possibility, as actualistic studies of catastrophic cetacean death assemblages show a wide variety of decay stages [42]. HAB-mediated mortalities at Cerro Ballena would also partly explain the absence of vertebrate scavenging and the absence of skeletal trauma [43]. The general completeness of rorqual skeletons, in contrast to the disarticulation of other marine vertebrates, reflects a size bias or temporal delay in scavenging, permitting more disarticulation and abrasion. Sharks represented by isolated teeth suggest attritional input or potential scavenging by-products (see the electronic supplementary material, figure S9). However, billfish remains (Xiphiidae and Istiophoridae) suggest that these large predatory consumers are similarly susceptible to HABs, a finding that has been reported in the modern world (see the electronic supplementary material). Isolated remains of aquatic sloths (*T. natans*) may reflect incidental, attritional input or actual HAB-mediated mortality based on extant HAB toxin transfers (i.e. inhalation) for modern herbivorous marine mammals [40]. Collectively, the taphonomy of Cerro Ballena indicates that repeated marine mammal mortalities were relatively rapid (hours to weeks in duration), geographically widespread and allochthonous (i.e. at sea). These latter traits are all consistent with HAB-related mortalities in the modern world, which show taphonomic signals that are temporally delayed and physically remote from their source [38,40,41,44].

The depositional environment in which vertebrate carcasses were buried was supratidal, based on ichnological and sedimentological evidence. We observed abundant traces of *Psilonichnus*, which typically occurs on supratidal flats, and *Skolithos* and *Ophiomorpha*, belonging to the Skolithos ichnofacies, also common to tidal flats (figure 2; see the electronic supplementary material, figure S10). In other parts of the fossil record, *Psilonichnus* has been interpreted as a crab trace fossil [45]. Given the unique food resource provided by marine mammal carcasses, it is not surprising to find scavenging traces on individual balaenopterid bones that we attribute to crabs (figure 2*e,f*). These short (approx. 1 cm), sharp and closely associated traces on the skull bones of MPC 662 are similar in trace morphology to those described for penguin bones from the Miocene of Argentina [46]. The lack of variation in grain size and scarcity of erosional surfaces in the section at Cerro Ballena further indicate a more or less constant sedimentation encompassing approximately 10–16 kyr of deposition, based on rates for modern tidal flats (see the electronic supplementary material). The preservation of delicate features resembling tufted algal mats (figure 2*c*, see [47] and the electronic supplementary material, figure S11) reflects rapid post-mortem replacement by iron oxides, and it also indicates the contemporaneous presence of algae and high iron concentrations, which promote algal growth in this depositional environment.

In terms of concentration mechanism, dead or dying marine vertebrates were delivered to south-facing embayment, protected from normal wave action by basement rocks and a barrier bar to the west (figure 1*b*). This interpretation is supported by the absence of north–south-oriented wave ripples (which should be present if this area had been directly exposed to Pacific Ocean waves) and the presence of low-angle planar cross-bedded sandstone at the top of the sequence, typical of a beach or berm (figure 2). Storm surges flooding the supratidal flats to a depth of about 1.5 m, as calculated from estimates of wind velocity, duration and fetch (see the electronic supplementary material), would have been sufficient to float the largest carcasses from the south, allowing hydraulic sorting to modally orient them at each level [48]. The absence of major disarticulation and limited skeletal scatter for any marine mammal skeleton further supports limited initial scavenging of floating carcasses, and rapid transit time (hours to days) between death at sea and coming to rest on a protected, supratidal flat. Such a shallow and mostly subaerial environment also excluded large marine scavengers. Equally, the surrounding desert environment (which already existed at the time) lacked sufficiently large terrestrial predators [49] that could dismember the largest of the carcasses.

Thus, the supratidal flat worked as a taphonomic trap [19], preserving carcasses that arrived during storms or spring tides in an excellent environment for decay *in situ*, mostly free of scavengers. As carcasses were buried under a relatively continuous rate of fine sediments, each mass stranding horizon yielded a discrete layer of skeletal remains. Sediment samples under light and scanning electron microscopy lacked distinct algal cell fragments (e.g. diatom frustules), but there were widespread approximately 5–10 μm spherical apatite grains encrusted in iron oxides (see the electronic supplementary material, figure S1), which could result from mineral replacement of non-siliceous algae (e.g. cyanobacteria) from coastal HABs. However, we cannot definitively confirm their biogenic source nor discriminate between their pre-depositional or post-depositional origins.

Alternative mass stranding death mechanisms lack modern analogues or fail to explain the full range of evidence at Cerro Ballena. For example, taxon-specific herding, breeding or stranding behaviours do not explain the full range of taxa at the site, which includes both pelagic and coastal species that do not inhabit supratidal environments (table 1). Tsunamis would have generated death assemblages lacking large body size selectivity (tables 1 and 2) and would result in high-energy sedimentary structures that are not present. Pandemic causes, such as morbillivirus, are not taxonomically broad, nor would it be parsimonious as a recurring mechanism over 10–16 kyr. Thus, all other alternative death mechanisms besides HABs fail to explain the iterative preservation of four bone-bearing levels.

The excavation quarry at Cerro Ballena yielded a density of associated, fossil marine mammal skeletons unrivaled elsewhere in the world. The density of individual cetacean specimens at Cerro Ballena, for example, is greater than other attritional deposits in the cetacean fossil record, including the Sharktooth Hill bonebed from the Middle Miocene of California [23] and the Eocene lagoonal deposits of Wadi Al-Hitan in Egypt [50]. The density of rorqual skeletons in BL1 (see the electronic supplementary material, table S12) alone is 10 times greater than associated, individual densities reported from the Mio-Pliocene Pisco Basin of southern Peru [51], which are preserved in distal, marine shelf environments, and notably lack multiple marine mammal species in close association (less than 10 m). Cerro Ballena is also surprising in its abundance of complete rorqual skeletons because baleen whale strandings are comparatively rare in the modern world. We propose that this ecological asymmetry arises from the shifted baseline of baleen whale abundances. In remote areas today, such as the Southern Ocean, there are examples of super-aggregations that are unusual by today's standards, but match historical and anecdotal accounts of baleen whale abundances prior to industrial whaling [52].

Evidence for HAB-mediated death assemblages of marine vertebrates in the fossil record is limited to only a few cases because of the difficulty in attributing HABs as a causal agent [53,54]. Modern analogues of marine mammal deaths caused by HABs outline a likely pathway that occurred repeatedly at this site during the Late Miocene. We propose that toxins, generated by HABs, poisoned multiple species of marine vertebrates, through ingestion of contaminated prey and/or inhalation, causing relatively rapid death at sea. Carcasses then floated towards the coastline, where they entered the estuary and were transported by locally generated, northward propagating storm waves into a restricted supratidal flat, where they were buried to the exclusion of major scavenging and disarticulation. This sequence was recorded four times during the deposition of sediment (approx. 10–16 kyr) at Cerro Ballena. The conditions that lead to this repeated phenomenon are tied to upwelling systems along westerly margins of continental coastlines [55]. Along the coast of western South America, ferruginous runoff from the Andes leads to increased iron in the ocean, which boosts productivity where iron is a limiting nutrient for phytoplankton growth [55], while also promoting HABs (e.g. cyanobacteria and dinoflagellates [44]). The antiquity of these processes probably pre-dates modern tectonic configurations, although it has not been documented in the fossil record until now. We propose that upwelling systems, fuelled by iron-rich runoff, in other regions of the world will have similar, repeated accumulations of marine consumers [56].
